# Use of FloSeal Sealant in the Surgical Management of Tubal Ectopic Pregnancy

**DOI:** 10.1155/2013/906825

**Published:** 2013-05-29

**Authors:** Mara Clapp, Jaou-Chen Huang

**Affiliations:** ^1^Department of Obstetrics and Gynecology, The University of Texas at Houston-Memorial Hermann, 6431 Fannin, Suite 3.214, Houston, TX 77030, USA; ^2^Department of Obstetrics and Gynecology, Texas Tech University Health Sciences Center, TTUHSC Mail Stop 8340, 3601 4th Street, Lubbock, TX 79430, USA

## Abstract

*Background*. Surgery is sometimes required for the management of tubal ectopic pregnancies. Historically, surgeons used electrosurgery to obtain hemostasis. Topical hemostatic sealants, such as FloSeal, may decrease the reliance on electrosurgery and reduce thermal injury to the tissue. *Case*. A 33-year-old G1 P0 received methotrexate for a right tubal pregnancy. The patient became symptomatic six days later and underwent a laparoscopic right salpingotomy. After multiple unsuccessful attempts to obtain hemostasis with electrocoagulation, FloSeal was used and hemostasis was obtained. Six weeks later, a hysterosalpingogram (HSG) confirmed tubal patency. The patient subsequently had an intrauterine pregnancy. *Conclusion*. FloSeal helped to achieve hemostasis during a laparoscopic salpingotomy and preserve tubal patency. FloSeal is an effective alternative and adjunct to electrosurgery in the surgical management of tubal pregnancy.

## 1. Introduction

Prior to the introduction of methotrexate, surgery was the only treatment option for tubal pregnancy. Methotrexate treatment can be highly successful in carefully selected patients. Moeller et al. conducted a randomized trial comparing methotrexate and laparoscopic surgery in the treatment of tubal pregnancies. They showed that methotrexate treatment had a success rate of 74%. However, 25% of patients receiving methotrexate required surgery [1]. Surgery is indicated in patients who are symptomatic (such as pain) and those who do not meet the criteria for medical therapy. Surgical management of a tubal pregnancy can either be a salpingotomy or a salpingectomy. A salpingotomy is the procedure of choice when patients desire future fertility.

Both mono- and bipolar electrosurgical instruments are utilized during surgery to obtain hemostasis. It is generally accepted that the former produces more thermal injury, because its energy has more lateral and vertical spread. Topical hemostatic agents have become an effective alternative to electrosurgery, because they do not require energy and, thus, produce no thermal injury. FloSeal (Baxter International Inc., Deerfield, IL) is a gelatin-thrombin topical sealant, which has been used in many surgical specialties, including neurosurgery, otolaryngology, cardiovascular, and gastrointestinal surgery [2–4]. Several publications described its use in ovarian cystectomies [5, 6]. However, the use of FloSeal in other gynecologic procedures, such as fertility-preserving laparoscopic salpingotomies, has not been reported.

## 2. Case Presentation

A 33-year-old G1 P0 Hispanic female originally presented for evaluation and management of infertility. Her relevant past medical history includes dysmenorrhea and no prior surgeries. She had no history of pelvic inflammatory disease or sexual transmitted diseases. An HSG showed a normal uterine cavity and bilateral tubal patency. A laparoscopy showed stage I endometriosis. Bilateral tubal patency was confirmed by chromotubation. A hysteroscopy showed an arcuate uterus. Semen analysis was normal.

The patient became pregnant after receiving Clomiphene superovulation and intrauterine insemination (IUI). However, the rise of human chorionic gonadotropin (hCG) was abnormal, and ultrasound showed no intrauterine pregnancy when the level of hCG crossed the threshold of detection (1200 mIU/mL). The patient elected for medical management and received 75 mg of methotrexate via intramuscular injection. The hCG level on the day of methotrexate administration (day one) was 858 mIU/mL. On day four, the hCG rose to 1376 mIU/mL. On day seven, the patient began to experience abdominal pain and vaginal bleeding. Physical examination showed stable vital signs, but the abdomen was diffusely tender with rebound pain. A transvaginal ultrasound showed free fluid in the cul-de-sac and a 16 × 16 mm mass in the right adnexa. The beta-hCG level at this time was 1314 mIU/mL. The patient was then taken to the operating room for a laparoscopic right salpingotomy.

**Figure 1:**
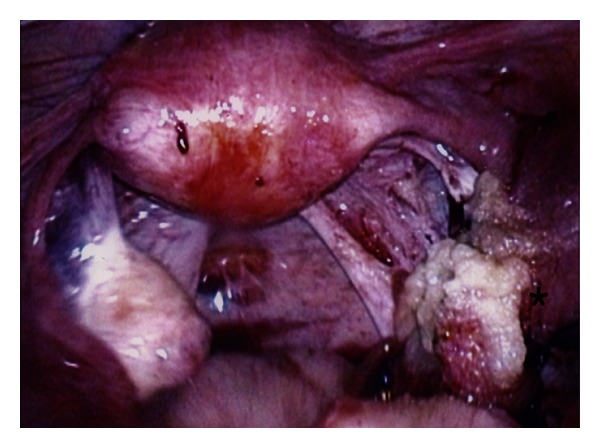
FloSeal is placed at the implantation site (*) for hemostasis during a right salpingotomy.

**Figure 2:**
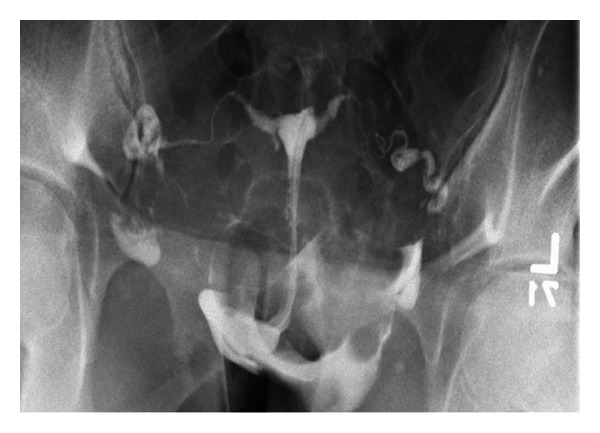
HSG reveals bilateral fallopian tube patency.

After the products of conception were removed using the hydrodissection technique, active bleeding was noted at the implantation site. The Kleppinger bipolar forceps were used to electrocoagulate the site multiple times but hemostasis was not achieved. FloSeal (5 mL) was then applied via an endoscopic adapter to the implantation site (see Figure 1). Hemostasis was obtained within a few minutes. Six weeks after surgery, the patient had a normal HSG, which confirmed bilateral tubal patency (see Figure 2).

The patient resumed treatments and achieved an intrauterine pregnancy on the fourth cycle of Clomiphene superovulation and IUI. An early ultrasound noted a viable intrauterine pregnancy, left corpus luteum, and normal adnexae bilaterally. Unfortunately, the pregnancy ended in a missed abortion requiring a suction dilation and curettage.

## 3. Discussion

Surgery is the only option when medical management of tubal pregnancy fails, as seen in our patient. It is generally accepted that, compared with salpingectomy, linear salpingotomy is associated with higher risks of persistent tubal pregnancy and recurrent ectopic pregnancy. Nonetheless, it is the preferred procedure in patients desiring future pregnancy [7].

A linear salpingotomy procedure involves making a 10–15 mm incision over the ectopic pregnancy at the antimesenteric side of the fallopian tube, and the products of conception are removed using the hydrodissection technique. Injecting diluted vasopressin at the site of the ectopic pregnancy prior to incision may decrease bleeding. Normally, bleeding at the implantation site is controlled by electrocoagulation or suture [7].

It is generally accepted that bipolar instruments produce less thermal injury than monopolar instruments due to decreased thermal spread. A recent study by Mohamed et al. indicates that bipolar electrocoagulation during a laparoscopic ovarian cystectomy adversely impacts ovarian reserve [8]. Although the impact of electrocoagulation on the function of the fallopian tube is unknown, it is reasonable to assume that less thermal damage is conducive to tubal patency and normal tubal function.

FloSeal (a gelatin-thrombin matrix hemostatic sealant) is a hemostatic agent of bovine origin. It was approved by the FDA in 1999 as an adjunctive hemostatic agent when conventional hemostatic agents are not effective during a surgical procedure. It is composed of two separate components, which are mixed prior to use. The first is a gelatin matrix, and the second is thrombin with a saline diluent. The two components act synergistically to form and stabilize a blood clot at the surgical site. The sealant is hydrophilic and works at wet, bleeding sites. Due to its liquid nature, it also conforms to the anatomy of the surgical site. The gelatin particles swell about 10–20% when they come in contact with blood, thus creating a pressure effect and a site for the intrinsic blood clot to build upon. Thrombin in the FloSeal helps convert the patient's fibrinogen into fibrin. Thrombin also helps activate Factor V, Factor VIII, and Factor XIII in the coagulation cascade. FloSeal is reabsorbed within 6 to 8 weeks [5].

FloSeal sealant has been used in many surgical specialties, including neurosurgery, otolaryngology, cardiovascular, and gastrointestinal surgery [2–4]. Several publications have advocated for its use in gynecologic surgery [5, 6]. Angioli et al. compared FloSeal with electrosurgery (bipolar forceps or carbon-dioxide laser) in the laparoscopic excision of endometriomas. They found no statistically significant difference between the two groups regarding time of hemostasis (median time for control group was 172 seconds and for FloSeal group was 182 seconds) [6]. The results suggest that FloSeal is a safe and effective alternative to electrosurgery in the setting of ovarian surgery. The additional benefit of FloSeal may include the avoidance of thermal damage. The use of FloSeal in tubal ectopic pregnancy and its impact on tubal patency have not been previously reported.

In summary, we used FloSeal to obtain hemostasis during a salpingotomy procedure, and tubal patency was preserved. While more data is needed, FloSeal is a promising hemostatic agent in the surgical management of tubal ectopic pregnancy.
